# Development, Implementation and Assessment of Molecular Diagnostics by Next Generation Sequencing in Personalized Treatment of Cancer: Experience of a Public Reference Healthcare Hospital

**DOI:** 10.3390/cancers11081196

**Published:** 2019-08-16

**Authors:** Javier Simarro, Rosa Murria, Gema Pérez-Simó, Marta Llop, Nuria Mancheño, David Ramos, Inmaculada de Juan, Eva Barragán, Begoña Laiz, Enrique Cases, Emilio Ansótegui, José Gómez-Codina, Jorge Aparicio, Carmen Salvador, Óscar Juan, Sarai Palanca

**Affiliations:** 1 Molecular Biology Unit, Service of Clinical Analysis, University and Polytechnic La Fe Hospital, 46026 Valencia, Spain; 2 Clinical and Translational Cancer Research Group, Health Research Institute La Fe, 46026 Valencia, Spain; 3 Department of Pathology, University and Polytechnic La Fe Hospital, 46026 Valencia, Spain; 4 Department of Pulmonology, University and Polytechnic La Fe Hospital, 46026 Valencia, Spain; 5 Department of Medical Oncology, University and Polytechnic La Fe Hospital, 46026 Valencia, Spain

**Keywords:** next generation sequencing, non-small cell lung cancer, metastatic colorectal cancer, molecular diagnostics, UNE-EN ISO 15189 accreditation

## Abstract

The establishment of precision medicine in cancer patients requires the study of several biomarkers. Single-gene testing approaches are limited by sample availability and turnaround time. Next generation sequencing (NGS) provides an alternative for detecting genetic alterations in several genes with low sample requirements. Here we show the implementation to routine diagnostics of a NGS assay under International Organization for Standardization (UNE-EN ISO 15189:2013) accreditation. For this purpose, 106 non-small cell lung cancer (NSCLC) and 102 metastatic colorectal cancer (mCRC) specimens were selected for NGS analysis with Oncomine Solid Tumor (ThermoFisher). In NSCLC the most prevalently mutated gene was *TP53* (49%), followed by *KRAS* (31%) and *EGFR* (13%); in mCRC, *TP53* (50%), *KRAS* (48%) and *PIK3CA* (16%) were the most frequently mutated genes. Moreover, NGS identified actionable genetic alterations in 58% of NSCLC patients, and 49% of mCRC patients did not harbor primary resistance mechanisms to anti-EGFR treatment. Validation with conventional approaches showed an overall agreement >90%. Turnaround time and cost analysis revealed that NGS implementation is feasible in the public healthcare context. Therefore, NGS is a multiplexed molecular diagnostic tool able to overcome the limitations of current molecular diagnosis in advanced cancer, allowing an improved and economically sustainable molecular profiling.

## 1. Introduction

Cancer is a complex and heterogeneous disease with considerable variation in histological and biological features. Understanding the role of genetic alterations involved in cancer development has led to its reclassification into different molecular subtypes that reflect biological behavior and may lead to further effective therapeutic targets to achieve improved outcome [1,2]. For this purpose, single gene testing approaches are traditionally used to identify individual alterations, currently targeted with approved drugs. However, comprehensive molecular characterization of tumors is hampered by the limited amount of cytology samples and/or formalin-fixed paraffin-embedded (FFPE) tissue biopsies, and by the turnaround time to assess multiple targetable genes and high economic costs. Moreover, the routine methods do not board the mutational co-occurrences, so they do not detect other alterations which in many cases are responsible for disease progression.

The knowledge of a tumor’s genetic profile is crucial to improve clinical-decision making in the patient management. Consequently, laboratories must integrate high-throughput sequencing technologies in routine molecular diagnostics [3]. These allow the simultaneous testing of multiple genetic alterations (point mutations, insertions, deletions, copy number variations and translocations) and quantify molecular subclones by procedures that provide accurate, reliable and cost-effective results. In this sense, next-generation sequencing (NGS) has overcome the cited challenges, posing an attractive alternative to traditional molecular diagnostic testing for cancer [4,5,6]. In fact, the College of American Pathologists has suggested the use of expanded panels in its latest guideline [7,8,9] and both the National Comprehensive Cancer Network (NCCN) and the European Society for Medical Oncology (ESMO) have proposed broader molecular profiling to identify rare driver mutations in non-small cell lung cancer (NSCLC) and metastatic colorectal cancer (mCRC) patients, for which effective drugs are already available or under development in clinical trials [10,11,12,13]. However, given the implications of NGS studies on the treatment of cancer patients, the establishment of an internal quality management system is strongly recommended. In this regard, the UNE-EN ISO 15189:2013 accreditation has been recognized as the international standard for quality management systems for all fields in clinical laboratories [14].

The purpose of the current study is to evaluate the integration of NGS technology in a routine clinical setting. We describe the mutational profile of two highly prevalent cancers (advanced NSCLC and mCRC) and analyze its diagnostic potential to characterize molecular heterogeneity and to increase the therapeutic opportunities with targeted therapies; we assess NGS technology at a technical and economical level; and we describe our experience in clinical practice of an NGS pipeline for cancer molecular diagnostics in the UNE-EN ISO 15189:2013 accreditation scope.

## 2. Results

### 2.1. Performance Evaluation of Next Generation Sequencing

#### 2.1.1. Next Generation Sequencing Quality Assessment

NGS assay was able to detect the seven low frequency variants (between 1–3%) present in two reference materials used as positive controls. The variant allele frequency (VAF) detected was consistent to the data obtained by digital droplet PCR (ddPCR) assays except for *EGFR* p.Leu858Arg and *KRAS* p.Gly13Asp mutations in which NGS VAF was slightly higher (5% versus 3% and 4% versus 3%, respectively).

Quality control analysis revealed excellent performance of the NGS panels (Figure S1). The median of total reads per sample was 347,362 with a median read depth of 3310 reads per amplicon. Uniformity was 97.1% on average and the average “on-target” reads per sample was 92.6%. Moreover, 96.6% of targeted bases showed ≥500 × read depth.

On the other hand, NGS showed an invalid test rate of 3.8% (8/208 FFPE specimens). Six NSCLC samples and two mCRC failed due to low sequencing quality metrics (total reads <100,000). These samples were subsequently excluded from the study.

#### 2.1.2. Comparison of Next Generation Sequencing with Conventional Methods

For *ROS1* rearrangements and *NRAS* mutations the overall agreement (OA) was 100% (Table 1). Regarding *EGFR*, one specimen showed the p.Thr790Met mutation by conventional methodology which was not reported by NGS (OA; 99.0%). Instead, NGS and Sanger Sequencing (SS) revealed a synonymous change in homozygosis in 787 codon p.(Gln787Gln). NGS technology allowed the detection of an *ALK* rearrangement not detected by immunohistochemistry (IHQ) or fluorescence in situ hybridization (FISH) (OA: 99.0%). In *KRAS,* seven specimens gave discordant results when comparing with Real Time (RT)-qPCR assay (OA: 94.7%). *BRAF* pVal600Glu mutation was detected by High Resolution Melting (HRM) in nine out of eleven NGS *BRAF* p.Val600Glu mutated samples (OA: 96.4%).

#### 2.1.3. Turnaround Time (TAT) and Cost Comparison

In order to compare NGS with conventional methodologies under theoretical conditions, we calculated the turnaround time and cost for three mandatory testing genes in NSCLC (*EGFR*, *ALK* and *ROS1*) and mCRC (*KRAS*, *NRAS* and *BRAF*). Starting in both cases from FFPE tissue blocks we were able to prepare libraries, sequence eight NSCLC or ten mCRC samples, and analyze data in five working days. Conventional methodologies for molecular testing of *EGFR*, *ALK* and *ROS1* resulted approximately in three days while testing *KRAS*, *NRAS* and *BRAF* in mCRC resulted approximately in four working days. However, if *KRAS* is positive, TAT is reduced to three days. TAT and cost comparison between NGS and conventional methods is shown in Table 2.

#### 2.1.4. Clinical Laboratory Accreditation

This thorough validation consisting of reference material analysis, quality control metrics assessment, experimental validation with conventional methodologies and TAT and cost comparison allowed us to include the assay within the scope of the recently granted UNE-EN ISO 15189:2013 accreditation (Entidad Nacional de Acreditación, ENAC, Nº1302/LE2445). Moreover, this NGS assay was externally validated within the European Genetics Quality Network (EMQN) External Quality Assessment Scheme for Oncogene Panel Testing, obtaining satisfactory results in 2017 and 2018 editions [15].

### 2.2. Next Generation Sequencing Results in the Routine Setting

#### 2.2.1. Pathogenic Alterations Detected by Next Generation Sequencing

**Figure 1:**
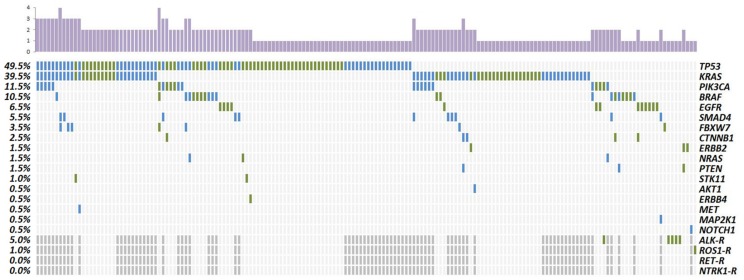
Distribution of gene alterations in NSCLC (green) and mCRC patients (blue). Column chart in the upper part represents the total number of mutations for each sample. Left column indicates the percentage of samples with specific gene alteration. Dark grey—Not tested. R—Rearrangements.

Sequencing analysis identified on average 1.45 non-synonymous and non-polymorphic variants per sample (291/200) (Figure 1). After filtering, a total of 168 different non-synonymous and non-polymorphic variants were detected, of which 149 were classified as somatic alterations previously reported and 19 were variants of uncertain significance (VUS).

In the NSCLC cohort, the most prevalently mutated gene was *TP53* (49%) followed by *KRAS* (31%), *EGFR* (13%), *BRAF* (11%) and *PIK3CA* (7%). Rearrangements were found in *ALK* and *ROS1* (5% and 1%, respectively). In the entire group, 9% of patients did not carry any somatic mutation; 56% harbored one somatic mutation and 35% two or more (Figure 1, Table S1). 

In mCRC patients, only 17% did not harbor any mutation, 33% carried one somatic mutation and 50% harbored two or more. Half of the patients carried mutations in *TP53* (50%). Mutations in *KRAS* were the second most prevalent (48%), specifically mutations in codon 12 accounted for 37%. We also detected pathogenic variants in codon 13 (5%), codon 146 (5%), codon 117 (1%) and the uncommon codon 19 mutation (p.Leu19Phe) found in concomitancy with a codon 146 mutation. Pathogenic variants in *PIK3CA* supposed 16% and mutations in *SMAD4* were detected in 11% of patients. Regarding *BRAF*, eight samples harbored the classical p.Val600Glu and two showed mutations outside this hotspot. *NRAS* mutated samples (2%) harbored the hotspot p.Gln61Arg mutation (Figure 1, Table S2). 

#### 2.2.2. Concurrent Molecular Alterations

**Figure 2:**
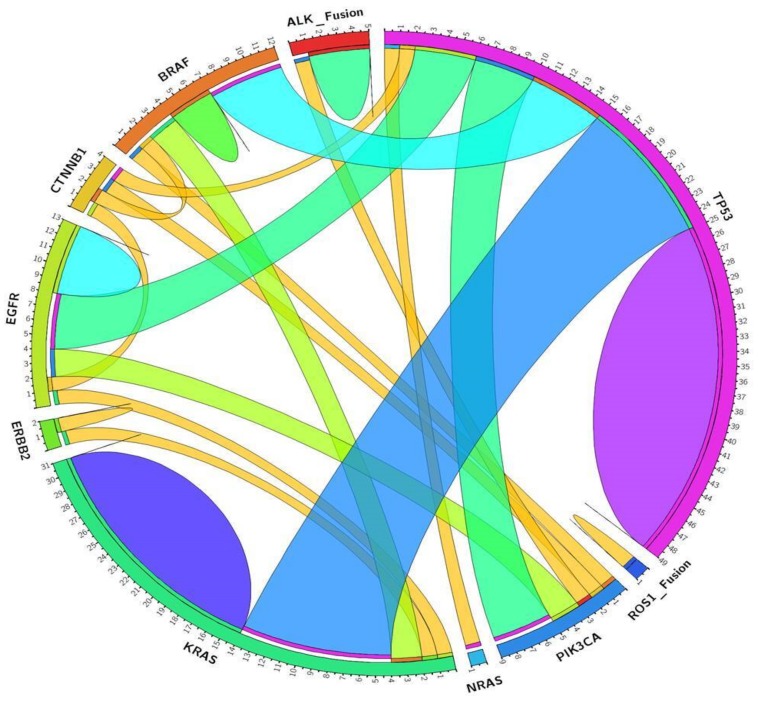
Circos diagram. Associations among the most prevalently mutated genes in NSCLC patients.

Thirty-five NSCLC patients harbored co-occurring mutations. One patient carried an indel in *EGFR* exon 19 accompanied by *KRAS* codon 12 mutation. Three *EGFR* mutations and one exon 19 deletion appeared in concurrency with *TP53* mutations. Two *EGFR* mutated patients also presented *PIK3CA* mutations; and one harbored a double *EGFR* codon 18 mutation in concurrency with *CTNNB1* mutation. Among the 31 *KRAS* mutated samples, two were in concurrency with *BRAF* mutations; one carried also a mutation in *ERBB2* and one sample harbored double *KRAS* mutations. One sample carrying a *NRAS* mutation also presented a *TP53* frameshift mutation. *PIK3CA* mutations were found to be concurrent with *EGFR*, *BRAF*, *TP53* mutations or *ALK* fusion. We identified two samples with three concurrent mutations. In one sample we identified mutations in *PIK3CA*, *CTNNB1* and *TP53*. Another patient carried mutations in *KRAS*, *STK11* and *TP53*. Moreover, one sample harbored four concurrent mutations in *PIK3CA*, *BRAF*, *FBXW7* and *TP53* (Figure 2, Table S1).

**Figure 3:**
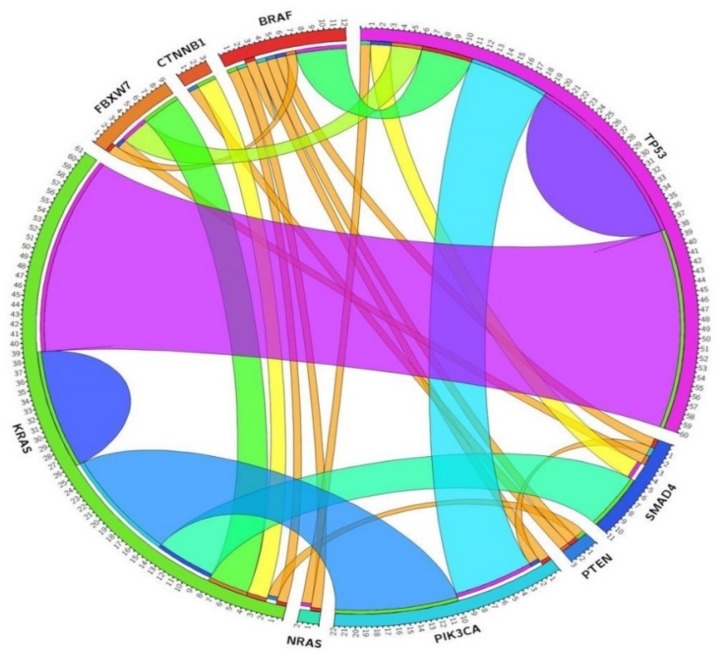
Circos diagram. Associations among the most prevalently mutated genes in mCRC patients.

Fifty mCRC patients harbored concurrent mutations. *KRAS* mutations were found to be concomitant with *TP53* (*n* = 11), *PIK3CA* (*n* = 5), *SMAD4* (*n* = 3) and *FBXW7* (*n* = 1), *CTNNB1* (*n* = 1) and *AKT* (*n* = 1). *BRAF* p.Val600Glu mutation was found in concurrency with *TP53* (*n* = 3), *PTEN* (*n* = 1), *PIK3CA* (*n* = 1) and *SMAD4* (*n* = 1). *NRAS* and *PIK3CA* were concomitant in one sample. Fifteen patients carried three concurrent mutations, *KRAS*-*PIK3CA*-*TP53* being the most frequent combination (*n* = 5). Interestingly, one patient harbored concurrent mutations in *KRAS*, *BRAF* and *TP53* and other carried mutations in *NRAS*, *BRAF* and *TP53*. One patient carried four concurrent mutations in *KRAS*, *SMAD4*, *FBXW7* and *TP53* (Figure 3, Table S2).

#### 2.2.3. Clinically Relevant Genetic Variants

**Figure 4:**
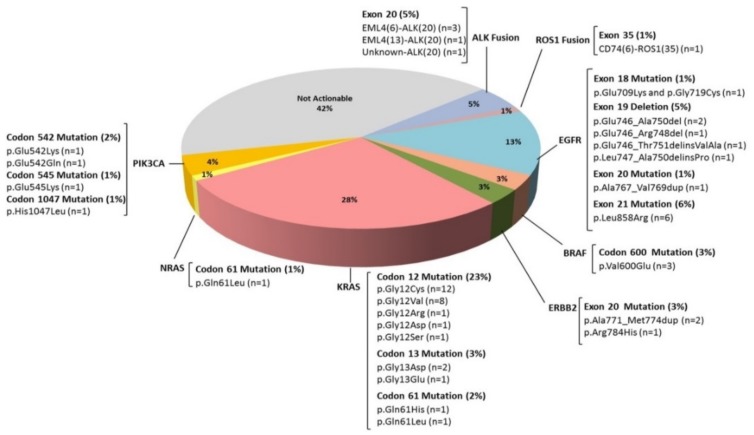
Percentage of NSCLC patients with actionable alterations detected by NGS. Fifty-eight percent of patients included in the study were susceptible to being treated with targeted drugs approved in advanced cancers or in clinical trials.

NGS identified actionable genomic alterations in 58% of NSCLC patients (Figure 4). The most prevalent changes detected in *EGFR* were exon 21 mutations (6%) followed by exon 19 alterations (5%). Codon 12 was the most frequently mutated in *KRAS* (23%) followed by codon 13 (3%) and codon 61 (2%). In *NRAS* only codon 61 was found mutated (1%). Regarding *BRAF*, three out eleven detected mutations occurred on the hotspot Val600. In regard to *PIK3CA*, codon 542 mutations were the most frequent (2%) followed by mutations in codons 545 and 1047 (1% for both). Duplication in exon 20 (p.Ala771_Met774dup; 2%) and the hotspot mutation p.Arg784His (1%) were found in *ERBB2*. Four patients showed fusions between *ALK* and *EML4*, in all of them, the rearrangement involved exon 20 of *ALK*, in three patients with exon 6 of *EML4* and in other with exon 13. One patient showed a fusion of *ALK* with an unknown partner. Finally, one patient presented a fusion between *ROS1* (exon 35) and *CD74* (exon 6).

**Figure 5:**
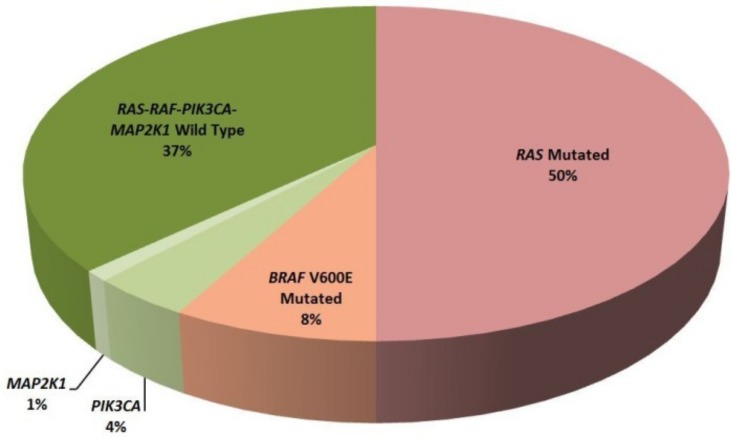
Classification of mCRC patients according to clinically relevant alterations detected by NGS.

Regarding targeted therapy in mCRC patients, 50% of patients harbored *RAS* mutations as a primary resistance mechanism to anti-EGFR therapies. Additionally, in the *RAS* wild type patients, NGS identified eight *BRAF* V600E mutated patients, five *PIK3CA* mutated patients and one patient harboring p.Lys57Asn in *MAP2K1* gene (Figure 5).

## 3. Discussion

NGS has emerged as a promising strategy to achieve precision medicine [16,17]. These approaches are able to identify multiple cancer genes simultaneously with low sample requirement, reducing sequencing costs and molecular diagnostics turn-around time. However, the integration a high-throughput technology into clinical routine practice of a public health system represents a major challenge. Laboratories performing clinically-relevant tests must improve their quality and competence [18] and for this purpose, accreditation and participation in External Quality Assessment (EQA) programs are strongly recommended [19]. 

In this study, we show that NGS technology is able to efficiently amplify and sequence multiple genes using only 10 ng of DNA or RNA obtained from FFPE samples. The quality metrics analysis revealed excellent read depth and coverage for all the targeted regions, allowing confident somatic variant detection [20]. These results technically validate the NGS assay and grant the identification of low VAF variants. The invalid test rate obtained (3.8%) is concordant with previously reported studies in FFPE samples [21,22].

In particular, these panels were able to confidently detect variants at <5% VAF, which can be relevant, in challenging, low tumor percentage FFPE samples. Therefore, and taking into account that current recommendations of NGS studies in FFPE samples propose not reporting variants with a VAF lower than 5% [23,24] (unless they have an important therapeutic or prognostic impact) we established this value as a threshold for reporting variants.

Moreover, we found an excellent correlation between NGS and single-gene conventional methods. The analysis of discordant results revealed that NGS is a more robust method compared with conventional approaches. Concerning *EGFR* mutations, one patient reported as positive with Cobas^®^ assay and negative by NGS also carried a homozygous and synonymous variant near codon 790 (p.Gln787Gln). However, since both NGS and SS did not detect this mutation, we hypothesize that the synonymous variant could affect primer or probe hybridization of the Cobas^®^ assay, resulting in a false positive detection of the p.Thr790Met mutation. In *KRAS* testing, we found seven discordant cases. Two samples resulted positive by RT-qPCR assay but were not detected by NGS. These samples were re-tested using a new lot of the AmoyDx assay, providing then concordant results with the NGS assay. Among the five negative samples for *KRAS* mutations by RT-qPCR, NGS reported mutations at low VAF in two cases (3.9% and 5.0%, respectively). In theory, these VAFs should be detected by the AmoyDx assay, which has a limit of detection (LOD) of 1–2%, established by using cell line DNA. However, we suspect this LOD could be higher when using highly degraded DNA obtained from FFPE samples. In the three remaining cases, NGS revealed *KRAS* mutations at high VAF (12%, 12% and 20%), which could be confirmed by SS. Moreover, these samples were re-tested by a technician in another institution, showing concordant results with the NGS assay. For *BRAF* p.Val600Glu mutation, NGS revealed two mutated samples with VAF of 4% and 5%, not detected by HRM (LOD = 10%). Regarding fusion transcripts, an OncoNetwork collaborative research study was able to detect *EML4*/*ALK* fusion up to 1% dilution [25]. In our cohort, we detected five fusions by NGS, of which one was not detected by IHQ nor FISH (53 nuclei counts). Our results are in agreement with Velizheva et al., who conclude that targeted NGS is a more robust and reliable method for fusion detection, especially in borderline cases, compared to single target assays such as FISH [26]. Taken together, NGS has proven to be a valid alternative to conventional molecular testing in terms of diagnostic accuracy [27].

TAT and economic costs are essential for NGS implementation in routine molecular diagnostics in a public healthcare hospital. Here, we found a great economic benefit in the employment of NGS technology versus conventional methodologies when it came to wild type *KRAS* mCRC patients. In *KRAS* mutated patients this benefit is not observed, however, a complete NGS test is achieved with a €30 difference per sample, therefore being an economically sustainable approach. In NSCLC, the extra cost associated with NGS studies (€51.5/patient) can be assumed based on the ability to identify actionable alterations with significant impact on patients’ outcome. 

The NGS approach described in this study requires a manual library and template preparation (emulsion PCR, enrichment and chip loading). Consequently, hands-on time is clearly higher than conventional studies in both NSCLC and mCRC samples. However, the development of new automatized devices for library and template preparation has drastically reduced hands-on time to approximately 1 hour, making NGS implementation in terms of technical staff much easier. Global time duration of NGS studies has also been higher than conventional approaches in NSCLC (5 versus 3 working days) and in mCRC (5 versus 3–4 working days depending on *KRAS* mutational status). This delay in molecular studies should not be an important limitation of NGS implementation because of its ability to identify clinically relevant alterations beyond the routinely tested genes. Moreover, the coexistence of both strategies may allow the choice of a faster conventional strategy when needed, especially in patients whose clinical situation requires a molecular result in a short period of time.

The implementation of this workflow in diagnostics routine was our first experience with NGS which allowed us to acquire a great expertise in amplicon-based NGS approaches. This has permitted us improve and optimized the process by implementing more complex gene panels (such as Oncomine Focus Assay, able to detect hotspot mutations in 35 genes, copy number variation in 19 genes and fusion transcripts of 23 driver genes) and automatizing the process (IonChef Instrument). Moreover, the development of new and faster sequencers (Ion S5 Instrument) is able to reduce global time duration to 4 days. However, it is important to acknowledge that NGS is economically sustainable when the appropriate number of samples is studied in the same experiment. In this sense, and according to the number of samples received for NGS studies, we are reporting NGS results under routine laboratory conditions in approximately 10–15 working days, as recommended [28,29,30].

The major advantage of the NGS approach is to provide information about potential therapeutic targets to improve clinical outcomes of patients with advanced cancer. Multiple biomarker testing has become a major challenge for molecular diagnostic laboratories because of the increasing number of approved targeted therapies and clinical trials. In this scenario NGS has been postulated as a technology with clinical applicability able to provide an exhaustive molecular profiling, deciphering tumoral heterogeneity that can in certain cases have a prognostic value and/or explain treatment resistance [31].

The mutation prevalence identified in our study for NSCLC and mCRC samples is concordant with previously published studies [32,33,34]. Exhaustive molecular profiling can provide relevant information for a patient’s clinical management. In our study, 58% of NSCLC patients harbored a potential clinically-actionable alteration, confirming the applicability of these studies for candidate selection. Regarding mCRC, in *RAS* wild type patients (*n* = 50) NGS identified four patients with mutated *PIK3CA*. Response to anti-EGFR treatment in these patients is still controversial [35] although *RAS*-*RAF* and *PIK3CA* wild-type patients seem to have better responses [36]. Moreover, one patient harbored the p.Lys57Asn mutation in *MAP2K1* that has been described as a primary resistance mechanism to anti-EGFR treatment [37]. Taken together NGS identified 49% of patients without resistance mechanisms to this targeted therapy.

Concurrent mutations have been detected in 35% of NSCLC patients and in 50% of mCRC patients revealing tumor biology complexity. Although there are no well-established molecular prognostic factors neither in NSCLC nor mCRC certain passenger mutations may be associated with an adverse prognosis. In this sense, *TP53* [38] or *STK11* in concomitancy with *KRAS* mutations [39] have been associated with a worse prognosis in NSCLC. In mCRC, *TP53* [40] or *SMAD4* [41] mutations have been related to a worse response to anti-EGFR therapy and *FBXW7* [42] has recently been described as a strong worse prognostic factor.

## 4. Materials and Methods 

### 4.1. Patients and Samples

The study included a series of 106 advanced NSCLC (stages III–IV) and 102 mCRC (stage IV) patients diagnosed in the Department of Medical Oncology at the University Hospital La Fe (Valencia, Spain) from 2015 to 2017. The epidemiological, clinical and pathological features of these patients are summarized in Table 3. All patients showed their agreement by signing the informed consent elaborated in accordance with the recommendations of the Declaration of Human Rights, the Conference of Helsinki [43] and the study was approved by the Hospital Ethics Committee (2015/0713; 16 February 2016, 2015/0096; 15 July 2016 2017/0070 29 March 2017), Tissue samples were examined in the Department of Pathology and only those with at least 150 total cells and 20% of tumor content were considered valid for molecular analysis. Two reference standard DNA samples provided by the European Molecular Genetics Quality Network (EMQN) were also used as positive controls.

### 4.2. DNA and RNA Preparation

Genomic DNA was isolated from three 5 μm thick FFPE sections using Deparaffinization Solution and the GeneRead DNA FFPE Kit (Qiagen, Hilden, Germany). RNA was extracted from three 15 μm thick FFPE sections employing the RecoverAllTM Total Nucleic Acid Isolation Kit (ThermoFisher Scientific, Waltham, MA, USA). DNA and RNA concentration was assessed using Qubit 3.0 fluorometer with DNA HS or RNA HS Assay Kit (ThermoFisher Scientific).

### 4.3. Molecular Analysis by Next Generation Sequencing

Molecular analysis was performed at the Molecular Biology Unit (University Hospital la Fe) using *Conformité Européenne*-In vitro diagnostic (CE-IVD) approved kits and workflows.

#### 4.3.1. Next Generation Sequencing Panels

Oncomine Solid Tumor DNA kit (OST-DNA; ThermoFisher Scientific) was used for mutation detection in 22 genes involved in colon and lung cancer (*AKT1* (NM_001014431.1), *ALK* (NM_004304.4), *BRAF* (NM_004333.4), *CTNNB1* (NM_001904.3), *DDR2* (NM_006182.2), *EGFR* (NM_005228.3), *ERBB2* (NM_004448.3), *ERBB4* (NM_005235.2), *FBXW7* (NM_033632.3), *FGFR1* (NM_001174067.1), *FGFR2* (NM_022970.3), *FGFR3* (NM_001163213.1), *KRAS* (NM_033360.3), *MAP2K1* (NM_002755.3), *MET* (NM_001127500.1), *NOTCH1* (NM_017617.3), *NRAS* (NM_002524.4), *PIK3CA* (NM_006218.2), *PTEN* (NM_000314.4), *SMAD4* (NM_005359.5), *STK11* (NM_000455.4), *TP53* (NM_000546.5)). The design includes 92 amplicons. For RNA sequencing of NSCLC samples, we used Oncomine Solid Tumor Fusion Transcript kit (OST-RNA; ThermoFisher Scientific), that allows the detection of fusion transcripts involving *ALK*, *RET*, *ROS1* and *NTRK1* genes with 85 amplicons. All NGS studies were conducted with the Ion Torrent Personal Genome Machine (PGM) technology (ThermoFisher Scientific).

#### 4.3.2. Ion Torrent Library Preparation

For DNA libraries preparation, multiplex PCR was performed on 10 ng of DNA. After primer digestion and barcode ligation, library fragments were purified with Agencourt^®^ AMPure^®^ XP (Beckman Coulter, Brea, CA, USA). Finally, quantification and dilution (100 pM) of the amplified libraries was performed using the Ion Library Equalizer Kit (ThermoFisher Scientific) as described by the manufacturer. 

RNA libraries preparation included a previous cDNA synthesis step from 10 ng of RNA using the SuperScript kit VILO cDNA synthesis kit (ThermoFisher Scientific). In this case, a multiplex PCR amplification of cDNA was performed. Library quantification was carried out by qPCR, inferring the concentration from a standard curve generated with Ion Library Quantification Kit (ThermoFisher Scientific). RNA libraries were diluted to a concentration of 100 pM.

In NSCLC, DNA and RNA libraries from eight patients were combined in a 4:1 proportion, generating the library pool. In mCRC samples, 10 DNA libraries were combined in equal proportion.

#### 4.3.3. Clonal Amplification and DNA Sequencing

The library pool was clonally amplified in an emulsion PCR reaction using Ion Sphere Particles (ISPs) in the One Touch 2 Instrument. Subsequently, template-positive ISPs were enriched using the Ion One Touch ES with the Ion PGM Hi-Q OT2 kit following manufacturer´s protocol. Enriched template-positive ISPs were subjected to sequencing on the Ion Torrent Personal Genome Machine (PGM) on a 318v2 Ion Chip using Ion PGM Sequencing Hi-Q kit (all kits from ThermoFisher Scientific). 

#### 4.3.4. Base Calling, Variant Annotation and Prediction Tools Analysis

Raw data processing and alignment to the hg19 human reference genome was performed with Torrent Suite v5.6. Aligned sequences (Binary Alignment Map (BAM) files) were automatically transferred to the Ion Reporter Software (v5.6) to perform variant calling/annotation by using commercial workflows. Intronic variants and synonymous changes were filtered out. Variants with low total read depth (<500 total) and/or low variant read depth (<20 reads) were excluded. Additionally, Variants were visually examined using the Integrative Genomics Viewer (IGV) software (v.2.4). Subsequently, sequence variation databases such as Catalogue of Somatic Mutations in Cancer (COSMIC) [44], VarSome [45], The 1000 Genomes Project [46] and Single Nucleotide Polymorphism Database (dbSNPs) [47] were used to assess the pathogenicity of the detected variants. In variants with unknown significance, prediction tools like Provean [48], Sorting intolerant from tolerant (SIFT) [49] and PolyPhen-2 [50] were used in order to predict the effect of the amino acid substitution on the protein structure and function.

### 4.4. Experimental Verification

Verification instead of a full validation analysis was performed according to our national accreditation body (ENAC; Entidad Nacional de Acreditación), since the OST-DNA and OST-RNA kits are CE-IVD approved. The performance of NGS testing was extensively evaluated on different aspects. Firstly, we used well-characterized reference material to assess the presence or absence of somatic variants (point mutation and small insertions/deletions) and their allele frequencies. Secondly, we considered the pre-analytical conditions and assessed the quality of NGS analysis on determining FFPE samples as start material, allowing us to establish the sequencing quality metrics. Thirdly, diagnostic sensitivity and specificity were determined experimentally by comparing it with conventional methods for routinely tested alterations; additionally, clinical reporting was adapted according to international diagnostic standards and professional guidelines. Finally, to ensure a consistent high standard of performance, it was essential to establish an EQA program to monitor the quality of NGS testing in clinical practice and to propose corrective actions when needed.

#### 4.4.1. Low Frequency Variant Detection

To evaluate the performance of the NGS assay for low frequency variant (<5%) detection we used reference materials provided by the European Molecular Genetics Quality Network (EMQN) in the External Quality Assessment Scheme for Oncogene Panel Testing (2017 and 2018). One of the reference materials used harbored the *EGFR* hotspot mutation p.Leu858Arg (VAF:3%), the *EGFR* deletion p.Glu746_Ala750del (VAF:2%) and the resistance hotspot mutation p.Thr790Met (VAF:1%) and the other harbored the following low frequency variants: *EGFR* p.Leu858Arg (VAF:3%), p.Thr790Met (VAF:2%), *KRAS* p.Gly13Asp (VAF:3%) and *PIK3CA* p.His1047Arg (VAF:3%). All described variants had previously been validated by ddPCR. 

#### 4.4.2. Next Generation Sequencing Metrics

The number of reads, mean depth, “on-target” reads and uniformity were the parameters used as quality control check points for further sample analysis. A total number of reads higher than 100,000 together with “on-target” and uniformity values >80% were required for each DNA library and 20,000 total reads for each RNA library.

#### 4.4.3. Assessment of the Diagnostic Sensitivity and Specificity of the NGS Assay

Detected missense mutations in *EGFR*, *NRAS*, *KRAS* and *BRAF* genes, as well as *ALK* and *ROS1* genes rearrangements were tested by conventional methods. *EGFR* mutations were validated by Cobas^®^ *EGFR* Mutation Test v2 (CE-IVD) (Roche Diagnostics, Basel, Switzerland); *NRAS* and *KRAS* mutations were confirmed by Real Time (RT)-qPCR using AmoyDx^®^ *KRAS* Mutation Detection Kit and AmoyDx^®^ *NRAS* Mutation Detection Kit (AmoyDx, Xiamen, China); *BRAF* mutations were validated by High Resolution Melting (HRM) as previously described [51]. *ALK* and *ROS1* rearrangements were studied by immunohistochemistry (IHQ) employing VENTANA *ALK* (D5F3) CDx Assay (Roche Diagnostics, Basel, Switzerland) and IHQ *ROS1* Clon D4D6 (Cell Signaling Technology, Danvers, MA, USA), respectively. Positive IHQ assays were confirmed by fluorescence in situ hybridization (FISH) employing Vysis *ALK* Break Apart FISH Probe Kit (Abbott Laboratories, Chicago, Illinois, USA) and Vysis 6q22 *ROS1* Break Apart FISH Probe Kit (Abbott Laboratories), respectively.

#### 4.4.4. External Quality Assessment (EQA) Program

To ensure a consistent high standard of performance, this assay was externally validated by the participation in the EMQN External Quality Assessment Scheme for Oncogene Panel Testing (2017/2018).

### 4.5. Statistics

Quantitative variables were summarized by their mean and standard deviation, and categorical variables by absolute frequencies. 

## 5. Conclusions

NGS is a technology able to assess multiple genetic biomarkers that has demonstrated a great concordance with conventional single target assays, providing an exhaustive molecular profiling of clinically relevant alterations at reasonable costs and turnaround times. The implementation of NGS in the diagnostic routine under the scope of UNE-EN ISO 15189:2013 accreditation has provided relevant information for patients’ clinical management, improving the molecular diagnostic in our center.

## Figures and Tables

**Table 1:** Comparison of NGS results with conventional methods.

Conventional Methods Result				
Gene	Mutation/Fusion Detected	Not Detected	Parameter	Agreement
*EGFR*-NGS Result				
Mutation Detected	13	0	PPA	92.9%
Not Detected	1	86	NPA	100%
			OA	99.0%
*KRAS*-NGS Result				
Mutation Detected	74	5	PPA	97.4%
Not Detected	2	50	NPA	90.9%
			OA	94.7%
*NRAS*-NGS Result				
Mutation Detected	3	0	PPA	100%
Not Detected	0	54	NPA	100%
			OA	100%
*BRAF*-NGS Result				
Mutation Detected	9	2	PPA	100%
Not Detected	0	45	NPA	95.8%
			OA	96.4%
*ALK* Fusions-NGS Result				
Fusion Detected	4	1	PPA	100%
Not Detected	0	95	NPA	99.0%
			OA	99.0%
*ROS1* Fusions-NGS Result				
Fusion Detected	1	0	PPA	100%
Not Detected	0	99	NPA	100%
			OA	100%

NGS—Next Generation Sequencing; PPA—Positive percent agreement; NPA—Negative percent agreement; OA—Overall agreement.

**Table 2:** Turnaround time and cost comparison between NGS and conventional methods.

Analysis	System	Hands-on Time, min (h)	Time Duration, min (h)	Costs (€)
**NGS Analysis (8 NSCLC samples (DNA + RNA))**				
DNA and RNA isolation	Manual	90 (1.5)	1140 (19.0)	166.96
Quantification and sample dilution	Qubit	30 (0.5)	30 (0.5)	13.68
Library preparation DNA	Veriti Thermal Cycler	120 (2.0)	1440 (24.0)	984.00
Library preparation RNA	Veriti Thermal Cycler			1214.72
Emulsion PCR	One Touch	20 (0.3)	480 (8.0)	124.16
Enrichment	One Touch ES	10 (0.2)	30 (0.5)	23.70
Sequencing	PGM System	10 (0.2)	240 (4.0)	607.00
Data Processing and analysis	Ion Reporter	160 (2.6)	180 (3.0)	-
Laboratory personnel costs †			-	235.60
Total Cost		-	-	3369.84
Cost per sample		-	-	421.23
Working days		440 (7.3)	5 days	-
**Conventional Molecular Analysis (8 NSCLC samples (DNA))**				
DNA isolation	Manual	60 (1.0)	1140 (19.0)	84.16
Quantification and sample dilution	Qubit	15 (0.25)	30 (0.5)	5.04
*EGFR* (Exon 18, 19, 20 and 21)	RT-qPCR	20 (0.3)	120 (2.0)	1391.50
*ALK*- Rearrangements	IHQ	10 (0.2)	960 (16.0)	672.00
*ROS1*-Rearrangements	IHQ	10 (0.2)	960 (16.0)	672.00
Sanger sequencing	SS	30 (0.5)	480 (8.0)	40.00
Data Processing and analysis		30 (0.5)	30 (0.5)	-
Laboratory personnel costs †			-	76.55
Total Cost		-	-	2941.27
Cost per sample				367.66
Working days		175 (2.9)	3 days	-
**NGS Analysis (10 mCRC samples (DNA))**				
DNA isolation	Manual	60 (1.0)	1140 (19.0)	105.20
Quantification and sample dilution	Qubit	15 (0.25)	30 (0.5)	6.30
Library preparation DNA	Veriti Thermal Cycler	120 (2.0)	1440 (24.0)	1230.00
Emulsion PCR	One Touch	20 (0.4)	480 (8.0)	77.60
Enrichment	One Touch ES	10 (0.2)	30 (0.5)	14.80
Sequencing	PGM System	10 (0.2)	240 (4.0)	379.40
Data Processing and analysis	Ion Reporter	160 (2.6)	180 (3.0)	-
Laboratory personnel costs †			-	219.85
Total Cost		-	-	2033.15
Cost per sample		-	-	203.32
Working days		395 (6.6)	5 days	-
**Conventional Molecular Analysis (10 mCRC samples (DNA))**				
DNA isolation	Manual	60 (1.0)	1140 (19.0)	105.20
Quantification and sample dilution	Qubit	15 (0.25)	30 (0.5)	6.30
*KRAS* (Exon 2,3 and 4)	RT-qPCR	20 (0.3)	150 (2.5)	1530.60
*NRAS* (Exon 2,3 and 4)	IHQ	20 (0.3)	150 (2.5)	1391.50
*BRAF* (Codon 600)	IHQ	30 (0.5)	120 (2.0)	1001.80
Sanger sequencing	SS	30 (0.5)	480 (8.0)	50.00
Data Processing and analysis		30 (0.5)	30 (0.5)	-
Laboratory personnel costs †		-	-	87.05
Total Cost		-	-	4172.47
Cost per sample		-	-	417.25
Working days		250 (3.4)	4 days *	-

NSCLC—Non-small cell lung cancer; mCRC—metastatic colorectal cancer; NGS—Next-generation sequencing; PGM—Personal Genome Machine; RT-qPCR—Real-Time quantitative polymerase chain reaction; SS—Sanger sequencing; IHQ—Immunohistochemistry; HRM—High resolution melting; - Non applicable. † Laboratory personnel costs—Cost is calculated based on the time required by the technician/physician in each analysis step (Hands-on time). * If *KRAS* is mutated global time duration has been estimated in 3 days.

**Table 3:** Epidemiological and clinical-pathological characteristics of the patients included.

NSCLC Patients (*n* = 100)		mCRC Patients (*n* = 100)	
Variable	*n*	Variable	*n*
Age (mean ±SD)	65.18 ± 10.66	Age (mean ±SD)	64.91 ± 10.82
Age, years		Age, years	
<60	30	<60	34
≥60	70	≥60	66
Gender		Gender	
Male	65	Male	63
Female	35	Female	37
Anatomic site		Anatomic site	
Primary tumor	85	Primary tumor	85
Regional lymph nodes	5	Liver	7
Brain	4	Lung	4
Liver	2	Peritoneum	2
Others	4	Others	2
Histologic NSCLC type		Histologic mCRC type	
Adenocarcinoma	87	Adenocarcinoma	100
Squamous Cell Carcinoma	3		
NOS	10		
Smoking status		Tumor Location	
Non-smoker	21	Sigmoid Colon	31
Ex-smoker	45	Rectum	26
Current-smoker	34	Right (ascending) colon	14
		Left (descending) colon	9
		Transverse colon	6
		Splenic flexure	5
		Cecum	3
		Unknown	6

NSCLC—Non-small cell lung cancer; mCRC—metastatic colorectal cancer; NOS—Not Otherwise Specified.

## Supplementary Materials

The following are available online at https://www.mdpi.com/2072-6694/11/8/1196/s1, Figure S1: NGS quality metrics, Table S1: Pathogenic variants detected in NSCLC patients, Table S2: Pathogenic variants detected in mCRC patients.
