# Supplementary material for: Development, Implementation and Assessment of Molecular Diagnostics by Next Generation Sequencing in Personalized Treatment of Cancer: Experience of a Public Reference Healthcare Hospital
Source: Cancers (Basel). 2019 Aug 16;11(8):1196. doi: 10.3390/cancers11081196 (PMC6721584; doi:10.3390/cancers11081196)
Supplement: Supplementary file 1 [file cancers-11-01196-s001.pdf]

Supplementary Materials: Development Implementation and Assessment of Molecular Diagnostics by Next Generation Sequencing in Personalized Treatment of Cancer: Experience of a Public Reference Healthcare Hospital.

Javier Simarro, Rosa Murria, Gema, Pérez-Simó, Marta Llop, Nuria Mancheño, David Ramos, Inmaculada de Juan, Eva Barragán, Begoña Laiz, Enrique Cases, Emilio Ansótegui, José Gómez-Codina, Jorge Aparicio, Carmen Salvador, Óscar Juan and Sarai Palanca

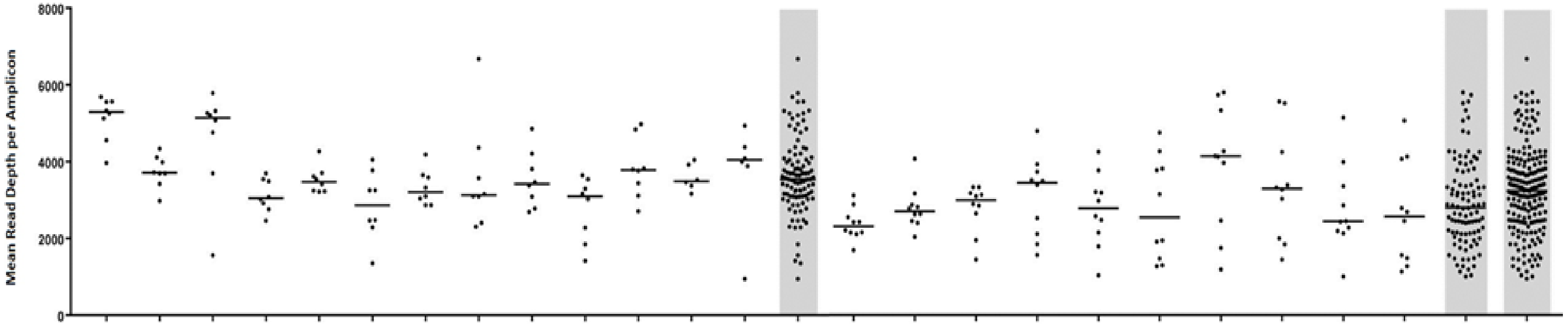

Run	1	2	3	4	5	6	7	8	9	10	11	12	13	ALL NSCLC
N	8	8	8	8	8	8	8	8	8	8	8	6	6	100
Read Depth	5298	3713	5144	3123	3478	2867	3215	3507	3424	3096	3782	3421	3945	3693
Total Reads	521,916	371,108	552,873	298,632	362,429	301,075	354,842	380,938	393,313	309,560	425,609	367,826	394,484	387,277
On Target (%)	95.91%	96.41%	91.49%	96.30%	94.03%	90.39%	90.90%	83.62%	84.48%	89.73%	91.56%	93.15%	96.96%	91.92%
Uniformity (%)	96.84%	98.90%	98.16%	99.50%	99.41%	99.41%	99.00%	95.48%	97.83%	97.72%	90.49%	96.31%	95.86%	97.30%

Run	1	2	3	4	5	6	7	8	9	10	ALL CRC	GLOBAL
N	10	10	10	10	10	10	10	10	10	10	100	200
Read Depth	2374	2788	2790	3094	2746	3407	3881	3366	2786	2027	2926	3310
Total Reads	244,990	300,911	300,654	319,646	290,499	352,862	410,780	341,904	291,217	220,924	307,448	347,362
On Target (%)	95.94%	91.07%	91.78%	92.73%	94.45%	92.96%	92.02%	94.96%	94.26%	93.05%	93.32%	92.62%
Uniformity (%)	95.70%	97.25%	97.94%	97.98%	97.72%	96.34%	98.79%	97.15%	95.63%	94.92%	96.94%	97.12%

Figure S1. NGS quality metrics. N- Number of samples analyzed per run; Read Depth- Represents the mean value of reads of each amplicon; Total Reads-Represent mean total reads per samples; “On target”-Represents the mean % of reads over the sequences of interest; Uniformity-Represents the mean % of uniformity coverage.

Table S1. Pathogenic variants detected in NSCLC patients.

Patient	Chr Position	Gene	NG/NM	Fusion	Mutation Type	Exon	Amino Acid Change	CDS	Total Coverage	Variant Coverage	VAF
NSCLC1	chr3:178952090	*PIK3CA*	NM_006218.2		missense	21	p.Gly1049Arg	c.3145G>C	3995	773	19 %
	chr7:55259515	*EGFR*	NM_005228.3		missense	21	p.Leu858Arg	c.2573T>G	3959	800	20 %
NSCLC2	chr7:55242462	*EGFR*	NM_005228.3		nonframeshift deletion	19	p.Glu746_Thr751delinsVA	c.2237_2253delinsTGCT	3996	2318	58 %
NSCLC3	chr7:55242464	*EGFR*	NM_005228.3		nonframeshift deletion	19	p.Glu746_Ala750del	c.2235_2249del	3535	107	3 %
NSCLC4	chr7:55259515	*EGFR*	NM_005228.3		missense	21	p.Leu858Arg	c.2573T>G	3523	390	11 %
NSCLC5	chr3:178952085	*PIK3CA*	NM_006218.2		missense	21	p.His1047Leu	c.3140A>T	3994	3052	76 %
	chr17:7578190	*TP53*	NM_000546.5		missense	6	p.Tyr220Cys	c.659A>G	3999	2653	66 %
NSCLC7	chr2:42522656	*ALK*	NG_009445.1	EML4 - ALK		6∣20					-
NSCLC8	chr17:7577094	*TP53*	NM_000546.5		missense	8	p.Arg282Trp	c.844C>T	1396	237	17 %
NSCLC9	chr12:25398284	*KRAS*	NM_033360.3		missense	2	p.Gly12Val	c.35G>T	3993	1375	34 %
	chr17:7578442	*TP53*	NM_000546.5		missense	5	p.Tyr163Cys	c.488A>G	1128	668	59 %
NSCLC10	chr17:7578457	*TP53*	NM_000546.5		missense	5	p.Arg158Pro	c.473G>C	1657	1365	82 %
NSCLC11	chr12:25380275	*KRAS*	NM_033360.3		missense	3	p.Gln61His	c.183A>T	3996	1568	39 %
NSCLC12	chr12:25398285	*KRAS*	NM_033360.3		missense	2	p.Gly12Cys	c.34G>T	3329	788	24 %
NSCLC13	chr12:25398284	*KRAS*	NM_033360.3		missense	2	p.Gly12Asp	c.35G>A	3996	1738	43 %
	chr17:7578530	*TP53*	NM_000546.5		missense	5	p.Phe134Val	c.400T>G	2745	743	27 %
NSCLC14	chr17:7577534	*TP53*	NM_000546.5		missense	7	p.Arg249Ser	c.747G>T	3215	535	17 %
	chr17:7578406	*TP53*	NM_000546.5		missense	5	p.Arg175Leu	c.524G>T	2127	211	10 %
NSCLC15	chr12:25398281	*KRAS*	NM_033360.3		missense	2	p.Gly13Asp	c.38G>A	3998	1275	32 %
	chr17:7578468	*TP53*	NM_000546.5		frameshift deletion	5	p.Gly154fs	c.461_461del	1119	228	20 %
NSCLC16	chr2:42522656	*ALK*	NG_009445.1	EML4 - ALK		6∣20					-
NSCLC17	chr3:41266113	*CTNNB1*	NM_001904.3		missense	3	p.Ser37Cys	c.110C>G	1781	298	17 %
	chr7:55241677	*EGFR*	NM_005228.3		missense	18	p.Glu709Lys	c.2125G>A	2721	1101	40 %
	chr7:55241707	*EGFR*	NM_005228.3		missense	18	p.Gly719Cys	c.2155G>T	2709	1100	41 %
NSCLC18	chr17:7577535	*TP53*	NM_000546.5		missense	7	p.Arg249Thr	c.746G>C	3997	1583	40 %
NSCLC19	chr7:55259515	*EGFR*	NM_005228.3		missense	21	p.Leu858Arg	c.2573T>G	3637	248	7 %
	chr17:7578269	*TP53*	NM_000546.5		missense	6	p.Leu194Phe	c.580C>T	2652	228	9 %
NSCLC20	chr12:25398281	*KRAS*	NM_033360.3		missense	2	p.Gly13Asp	c.38G>A	3513	599	17 %
	chr17:7578461	*TP53*	NM_000546.5		missense	5	p.Val157Phe	c.469G>T	3379	849	25 %
NSCLC22	chr6:117645578	*ROS1*	NG_033929.1	EZR - ROS1		10∣34					-
NSCLC23	chr7:140453136	*BRAF*	NM_004333.4		missense	15	p.Val600Glu	c.1799T>A	3997	128	3 %
NSCLC24	chr7:140453136	*BRAF*	NM_004333.4		missense	15	p.Val600Glu	c.1799T>A	3993	205	5 %
	chr12:25380275	*KRAS*	NM_033360.3		missense	3	p.Gln61His	c.183A>C	3996	525	13 %
NSCLC25	chr12:25398285	*KRAS*	NM_033360.3		missense	2	p.Gly12Cys	c.34G>T	3994	248	6 %
NSCLC26	chr12:25398284	*KRAS*	NM_033360.3		missense	2	p.Gly12Val	c.35G>T	3988	305	8 %
	chr17:7578535	*TP53*	NM_000546.5		missense	5	p.Lys132Arg	c.395A>G	3098	176	6 %
NSCLC27	chr12:25398284	*KRAS*	NM_033360.3		missense	2	p.Gly12Val	c.35G>T	3880	504	13 %
NSCLC28	chr7:140481402	*BRAF*	NM_004333.4		missense	11	p.Gly469Val	c.1406G>T	3956	1038	26 %
NSCLC29	chr17:7577538	*TP53*	NM_000546.5		missense	7	p.Arg248Leu	c.743G>T	3958	669	17 %
NSCLC30	chr12:25398285	*KRAS*	NM_033360.3		missense	2	p.Gly12Cys	c.34G>T	2386	357	15 %
NSCLC31	chr12:25398284	*KRAS*	NM_033360.3		missense	2	p.Gly12Val	c.35G>T	2688	738	27 %
	chr17:7577538	*TP53*	NM_000546.5		missense	7	p.Arg248Leu	c.743G>T	3878	629	16 %

	chr19:1221313	*STK11*	NM_000455.4		frameshift deletion	6	p.Pro281fs	c.837_837del	1984	567	29 %
NSCLC32	chr12:25398284	*KRAS*	NM_033360.3		missense	2	p.Gly12Val	c.35G>T	3905	976	25 %
NSCLC33	chr17:7578469	*TP53*	NM_000546.5		missense	5	p.Gly154Val	c.461G>T	1336	469	35 %
NSCLC34	chr17:7574003	*TP53*	NM_000546.5		nonsense	10	p.Arg342Ter	c.1024C>T	3695	763	21 %
	chr17:7577114	*TP53*	NM_000546.5		frameshift insertion	8	p.Cys275fs	c.823_824insT	2190	87	4 %
NSCLC35	chr12:25398285	*KRAS*	NM_033360.3		missense	2	p.Gly12Cys	c.34G>T	3999	2387	60 %
	chr17:7577120	*TP53*	NM_000546.5		missense	8	p.Arg273Leu	c.818G>T	1726	900	52 %
NSCLC36	chr17:7578403	*TP53*	NM_000546.5		missense	5	p.Cys176Tyr	c.527G>A	2573	1840	72 %
NSCLC38	chr12:25398285	*KRAS*	NM_033360.3		missense	2	p.Gly12Cys	c.34G>T	2941	254	9 %
NSCLC39	chr3:41266134	*CTNNB1*	NM_001904.3		missense	3	p.Pro44Leu	c.131C>T	2260	789	35 %
	chr3:178936091	*PIK3CA*	NM_006218.2		missense	10	p.Glu545Lys	c.1633G>A	3887	550	14 %
	chr17:7579355	*TP53*	NM_000546.5		missense	4	p.Leu111Arg	c.332T>G	2014	785	39 %
NSCLC40	chr7:140453134	*BRAF*	NM_004333.4		missense	15	p.Lys601Glu	c.1801A>G	1452	397	27 %
NSCLC41	chr17:7577046	*TP53*	NM_000546.5		nonsense	8	p.Glu298Ter	c.892G>T	1550	145	9 %
NSCLC42	chr17:7579358	*TP53*	NM_000546.5		missense	4	p.Arg110Leu	c.329G>T	552	130	24 %
NSCLC43	chr12:25398284	*KRAS*	NM_033360.3		missense	2	p.Gly12Val	c.35G>T	2833	170	6 %
NSCLC44	chr17:7578526	*TP53*	NM_000546.5		missense	5	p.Cys135Tyr	c.404G>A	1405	638	45 %
NSCLC45	chr17:7578208	*TP53*	NM_000546.5		missense	6	p.His214Arg	c.641A>G	3215	1476	46 %
NSCLC46	chr12:25398284	*KRAS*	NM_033360.3		missense	2	p.Gly12Val	c.35G>T	3996	896	22 %
NSCLC48	chr3:178936082	*PIK3CA*	NM_006218.2		missense	10	p.Glu542Gln	c.1624G>C	3664	282	8 %
	chr17:7578461	*TP53*	NM_000546.5		missense	5	p.Val157Phe	c.469G>T	2290	225	10 %
NSCLC49	chr17:7578395	*TP53*	NM_000546.5		missense	5	p.His179Tyr	c.535C>T	3965	845	21 %
NSCLC50	chr12:25398285	*KRAS*	NM_033360.3		missense	2	p.Gly12Cys	c.34G>T	3991	1456	36 %
	chr17:7577528	*TP53*	NM_000546.5		nonframeshift deletion	7	p.Arg249del	c.745_747delAGG	3912	1240	32 %
NSCLC51	chr12:25398285	*KRAS*	NM_033360.3		missense	2	p.Gly12Cys	c.34G>T	3728	888	24 %
	chr17:7578517	*TP53*	NM_000546.5		missense	5	p.Ala138Val	c.413C>T	2464	486	20 %
NSCLC52	chr7:140453154	*BRAF*	NM_004333.4		missense	15	p.Asp594Val	c.1781A>T	1493	64	4 %
	chr12:25398281	*KRAS*	NM_033360.3		missense	2	p.Gly13Asp	c.38G>A	1946	98	5 %
NSCLC53	chr10:89717712	*PTEN*	NM_000314.4		missense	7	p.Pro246Leu	c.737C>T	3977	422	11 %
	chr17:37880981	*ERBB2*	NM_004448.3		duplication	20	p.Ala771_Met774dup	c.2311_2322dup	2146	556	26 %
NSCLC55	chr17:7577022	*TP53*	NM_000546.5		nonsense	8	p.Arg306Ter	c.916C>T	1091	300	28 %
NSCLC56	chr4:153247289	*FBXW7*	NM_033632.3		missense	10	p.Arg505Ser	c.1513C>A	3996	121	3 %
NSCLC57	chr7:55242478	*EGFR*	NM_005228.3		frameshift deletion		p.Glu747_Ala750delinsP	c.2238_2248delinsGC	3559	172	5 %
	chr12:25398285	*KRAS*	NM_033360.3		missense	2	p.Gly12Cys	c.34G>T	3438	2740	80 %
NSCLC58	chr7:140481411	*BRAF*	NM_004333.4		missense	11	p.Gly466Val	c.1397G>T	2459	453	18 %
	chr17:7579882	*TP53*	NM_000546.5		nonsense	2	p.Glu11Ter	c.31G>T	3442	630	18 %
NSCLC59	chr7:55242468	*EGFR*	NM_005228.3		nonframeshift deletion	19	p.Glu746_Arg748del	c.2236_2244del	1647	1101	67 %
	chr17:7577105	*TP53*	NM_000546.5		missense	8	p.Pro278Arg	c.833C>G	524	84	16 %
NSCLC60	chr2:42522656	*ALK*	NG_009445.1	EML4 ǀ ALK		13 ǀ 20					-
NSCLC61	chr7:140453136	*BRAF*	NM_004333.4		missense	15	p.Val600Glu	c.1799T>A	3920	227	6 %
	chr17:7577517	*TP53*	NM_000546.5		missense	7	p.Ile255Asn	c.764T>A	1850	104	6 %
NSCLC64	chr1:115256529	*NRAS*	NM_002524.4		missense	3	p.Gln61Leu	c.182A>T	3987	500	13 %
	chr17:7577035	*TP53*	NM_000546.5		frameshift deletion	8	p.Pro301fs	c.902_902delC	1416	89	6 %
NSCLC65	chr3:178952146	*PIK3CA*	NM_006218.2		missense	21	p.Leu1067Phe	c.3201G>C	3997	365	9 %
	chr4:153249460	*FBXW7*	NM_033632.3		missense	9	p.Asp440His	c.1318G>C	3996	263	7 %
	chr7:140481411	*BRAF*	NM_004333.4		missense	11	p.Gly466Ala	c.1397G>C	2599	651	25 %

	chr17:7578441	*TP53*	NM_000546.5		nonsense	5	p.Tyr163Ter	c.489C>A	1795	444	25 %
NSCLC66	chr2:42522656	*ALK*	NG_009445.1	EML4 - ALK		6|20					-
NSCLC67	chr17:7578452	*TP53*	NM_000546.5		frameshift deletion	5	p.Met160fs	c.477_477delC	3261	579	18 %
NSCLC68	chr7:55259515	*EGFR*	NM_005228.3		missense	21	p.Leu858Arg	c.2573T>G	3973	113	3 %
	chr17:7578430	*TP53*	NM_000546.5		frameshift deletion	5	p.Gln167fs	c.499_499delC	3289	170	5 %
NSCLC69	chr17:7578280	*TP53*	NM_000546.5		missense	6	p.Pro190Leu	c.569C>T	1334	114	9 %
NSCLC70	chr12:25398280	*KRAS*	NM_033360.3		missense	2	p.Gly13Glu	c.38_39delinsAA	3982	1797	45 %
NSCLC71	chr17:7577099	*TP53*	NM_000546.5		missense	8	p.Arg280Thr	c.839G>C	1332	461	35 %
NSCLC72	chr17:7577539	*TP53*	NM_000546.5		missense	7	p.Arg248Trp	c.742C>T	1676	616	37 %
NSCLC73	chr17:7577108	*TP53*	NM_000546.5		missense	8	p.Cys277Phe	c.830G>T	1465	473	32 %
NSCLC74	chr7:55259515	*EGFR*	NM_005228.3		missense	21	p.Leu858Arg	c.2573T>G	523	167	32 %
NSCLC75	chr12:25398285	*KRAS*	NM_033360.3		missense	2	p.Gly12Cys	c.34G>T	3998	641	16 %
NSCLC76	chr12:25398285	*KRAS*	NM_033360.3		missense	2	p.Gly12Cys	c.34G>T	3994	2031	51 %
	chr17:7577534	*TP53*	NM_000546.5		missense	7	p.Arg249Ser	c.747G>C	1786	642	36 %
NSCLC78	chr12:25380285	*KRAS*	NM_033360.3		missense	3	p.Thr58Ile	c.173C>T	4000	98	2 %
	chr12:25398285	*KRAS*	NM_033360.3		missense	2	p.Gly12Cys	c.34G>T	3994	1132	28 %
NSCLC79	chr7:140481411	*BRAF*	NM_004333.4		missense	11	p.Gly466Val	c.1397G>T	3999	1994	50 %
	chr17:7577103	*TP53*	NM_000546.5		frameshift insertion	8	p.Gly279fs	c.834_835insT	2670	1151	43 %
NSCLC80	chr2:212578301	*ERBB4*	NM_005235.2		missense	8	p.Gly319Val	c.956G>T	2793	1008	36 %
	chr17:7578457	*TP53*	NM_000546.5		missense	5	p.Arg158Leu	c.473G>T	1036	784	76 %
NSCLC81	chr17:37880981	*ERBB2*	NM_004448.3		duplication	20	p.Ala771_Met774dup	c.2311_2322dup	1467	200	14 %
NSCLC82	chr7:140481411	*BRAF*	NM_004333.4		missense	11	p.Gly466Val	c.1397G>T	3993	343	9 %
	chr17:7578442	*TP53*	NM_000546.5		missense	5	p.Tyr163Cys	c.488A>G	3860	238	6 %
NSCLC83	chr12:25398285	*KRAS*	NM_033360.3		missense	2	p.Gly12Arg	c.34G>C	3993	777	19 %
NSCLC84	chr17:7578469	*TP53*	NM_000546.5		missense	5	p.Gly154Val	c.461G>T	1480	351	24 %
NSCLC85	chr17:7578392	*TP53*	NM_000546.5		nonsense	5	p.Glu180Ter	c.538G>T	1666	534	32 %
	chr19:1220487	*STK11*	NM_000455.4		missense	4	p.Asp194Tyr	c.580G>T	749	209	28 %
NSCLC86	chr17:7577090	*TP53*	NM_000546.5		missense	8	p.Arg283Pro	c.848G>C	1148	51	4 %
NSCLC87	chr17:7578431	*TP53*	NM_000546.5		nonsense	5	p.Gln167Ter	c.499C>T	1896	880	46 %
NSCLC88	chr3:41266098	*CTNNB1*	NM_001904.3		missense	3	p.Asp32Val	c.95A>T	513	20	4 %
	chr7:140453155	*BRAF*	NM_004333.4		missense	15	p.Asp594Asn	c.1780G>A	798	187	23 %
NSCLC89	chr3:178936082	*PIK3CA*	NM_006218.2		missense	10	p.Glu542Lys	c.1624 G>A	710	16	2 %
	chr7:55242464	*EGFR*	NM_005228.3		nonframeshift deletion	19	p.Glu746_Ala750del	c.2235_2249del	1283	242	19 %
NSCLC90	chr12:25398284	*KRAS*	NM_033360.3		missense	2	p.Gly12Val	c.35G>T	3993	589	15 %
NSCLC91	chr12:25398285	*KRAS*	NM_033360.3		missense	2	p.Gly12Cys	c.34G>T	3996	157	4 %
	chr17:37881022	*ERBB2*	NM_004448.3		missense	20	p.Arg784His	c.2351G>A	4000	147	4 %
NSCLC92	chr7:55259515	*EGFR*	NM_005228.3		missense	21	p.Leu858Arg	c.2573T>G	3989	409	10 %
	chr17:7578469	*TP53*	NM_000546.5		missense	5	p.Gly154Val	c.461G>T	2903	199	7 %
NSCLC93	chr7:55248998	*EGFR*	NM_005228.3		duplication	20	p.Ala767_Val769dup	c.2300_2308dup	2758	1009	37 %
NSCLC94	chr12:25380276	*KRAS*	NM_033360.3		missense	3	p.Gln61Leu	c.182A>T	3998	535	13 %
NSCLC96	chr12:25398285	*KRAS*	NM_033360.3		missense	2	p.Gly12Ser	c.34G>A	1552	287	18 %
NSCLC97	chr2:42522656	*ALK*	NG_009445.1	ALK Fusion							-
	chr3:178936082	*PIK3CA*	NM_006218.2		missense	9	p.Glu542Lys	c.1624G>A	1994	136	7 %
NSCLC98	chr17:7578394	*TP53*	NM_000546.5		missense	5	p.His179Arg	c.536A>G	1769	198	11 %
NSCLC99	chr12:25398285	*KRAS*	NM_033360.3		missense	2	p.Gly12Cys	c.34G>T	3659	509	14 %
NSCLC100	chr17:7578457	*TP53*	NM_000546.5		missense	5	p.Arg158Leu	c.473G>T	3835	2682	70 %

CDS- Coding sequence nomenclature; Total Coverage- Total reads in the genomic region; Variant Coverage- Total variant reads; VAF- Variant allele frequency (%).

Table S2. Pathogenic variants detected in mCRC patients.

Patient	Chr Position	Gene	NG/NM	Mutation Type	Exon	Amino Acid Change	CDS	Total Coverage	Variant Coverage	VAF
CRC1	chr17:7577547	*TP53*	NM_000546.5	Missense	7	p.Gly245Val	c.734G>T	1686	166	10 %
CRC3	chr12:25398284	*KRAS*	NM_033360.3	Missense	2	p.Gly12Asp	c.35G>A	4777	1603	34 %
	chr18:48591826	*SMAD4*	NM_005359.5	Missense	9	p.Glu330Gly	c.989A>G	3709	1556	42 %
CRC4	chr12:25398285	*KRAS*	NM_033360.3	Missense	2	p.Gly12Cys	c.34G>T	2505	547	22 %
	chr3:178936082	*PIK3CA*	NM_006218.2	Missense	9	p.Gly542Lys	c.1624 G>A	3142	705	22 %
CRC5	chr12:25398285	*KRAS*	NM_033360.3	Missense	2	p.Gly12Cys	c.34G>T	3997	1126	28 %
	chr17:7578382	*TP53*	NM_000546.5	Nonsense	5	p.Ser183Ter	c.548C>G	2085	993	48 %
CRC6	chr17:7578392	*TP53*	NM_000546.5	Deletion	5	p. Arg174_His179del	c.520_537del	1490	730	49 %
	chr17:7578269	*TP53*	NM_000546.5	Missense	6	p.Leu194Phe	c.580C>T	1846	49	3 %
CRC7	chr12:25398284	*KRAS*	NM_033360.3	Missense	2	p.Gly12Val	c.35G>T	1544	236	15 %
CRC8	chr12:25398284	*KRAS*	NM_033360.3	Missense	2	p.Gly12Asp	c.35G>A	3994	1.358	34 %
CRC9	chr12:25398284	*KRAS*	NM_033360.3	Missense	2	p.Gly12Asp	c.35G>A	2733	1.613	59 %
CRC10	chr3:178936082	*PIK3CA*	NM_006218.2	Missense	9	p.Gly542Lys	c.1624 G>A	3689	165	4 %
	chr17:7577120	*TP53*	NM_000546.5	Missense	8	p.Arg273His	c.818G>A	1433	166	12 %
CRC11	chr17:7578212	*TP53*	NM_000546.5	Nonsense	6	p.Arg213Ter	c.637C>T	2045	382	19 %
CRC12	chr12:25398285	*KRAS*	NM_033360.3	Missense	2	p.Gly12Cys	c.34G>T	3997	1.126	28 %
	chr3:178936082	*PIK3CA*	NM_006218.2	Missense	9	p.Gly542Lys	c.1624 G>A	2268	58	3 %
	chr17:7577121	*TP53*	NM_000546.5	Missense	8	p.Arg273Cys	c.817C>T	2150	163	8 %
CRC13	chr12:25398284	*KRAS*	NM_033360.3	Missense	2	p.Gly12Ala	c.35G>C	2938	762	26 %
	chr17:7578395	*TP53*	NM_000546.5	Missense	5	p.His179Tyr	c.535C>T	2444	1.040	43 %
CRC14	chr12:25398284	*KRAS*	NM_033360.3	Missense	2	p.Gly12Val	c.35G>T	2870	207	7 %
	chr3:178952090	*PIK3CA*	NM_006218.2	Missense	20	p.His1049Arg	c.3145G>C	3123	226	7 %
	chr17:7578406	*TP53*	NM_000546.5	Missense	5	p.Arg175His	c.524G>A	2765	144	5 %
CRC15	chr1:115256529	*NRAS*	NM_002524.4	Missense	3	p.Gln61Arg	c.182A>G	1773	105	6 %
	chr3:178936082	*PIK3CA*	NM_006218.2	Missense	9	p.Gly542Lys	c.1624 G>A	1847	107	6 %
CRC16	chr7:140453136	*BRAF*	NM_004333.4	Missense	15	p.Val600Glu	c.1799T>A	2154	356	17 %
CRC18	chr12:25378647	*KRAS*	NM_033360.3	Missense	4	p.Lys117Asn	c.351A>T	3897	1.888	48 %
CRC19	chr17:7577538	*TP53*	NM_000546.5	Missense	7	p.Arg248Gln	c.743G>A	3984	330	8 %
CRC20	chr12:25398284	*KRAS*	NM_033360.3	Missense	2	p.Gly12Asp	c.35G>A	3993	710	18 %
	chr3:178936082	*PIK3CA*	NM_006218.2	Missense	9	p.Gly542Lys	c.1624 G>A	3930	100	3 %
	chr17:7577538	*TP53*	NM_000546.5	Missense	7	p.Arg248Gln	c.743G>A	3974	923	23 %
CRC21	chr7:140453136	*BRAF*	NM_004333.4	Missense	15	p.Val600Glu	c.1799T>A	3987	1.237	31 %
	chr17:7577022	*TP53*	NM_000546.5	Nonsense	8	p.Arg306Ter	c.916C>T	1148	515	45 %
CRC22	chr3:178936095	*PIK3CA*	NM_006218.2	Missense	9	p.Gln546Arg	c.1637A>G	3410	310	9 %
	chr18:48603032	*SMAD4*	NM_005359.5	Nonsense	11	p.Arg445Ter	c.1333C>T	2530	169	7 %
	chr17:7578403	*TP53*	NM_000546.5	Missense	5	p.Cys176Tyr	c.527G>A	553	110	20 %
CRC23	chr3:178952085	*PIK3CA*	NM_006218.2	Missense	20	p.His1047Arg	c.3140A>G	1384	286	21 %
	chr17:7577539	*TP53*	NM_000546.5	Missense	7	p.Arg248Trp	c.742C>T	1811	260	14 %
CRC24	chr7:140453136	*BRAF*	NM_004333.4	Missense	15	p.Val600Glu	c.1799T>A	2192	314	14 %
	chr4:153249348	*FBXW7*	NM_033632.3	Missense	8	p.Arg465His	c.1394G>A	3283	765	23 %

	chr17:7577547	*TP53*	NM_000546.5	Missense	7	p.Gly245Asp	c.734G>A	1304	252	19 %
CRC27	chr12:25398285	*KRAS*	NM_033360.3	Missense	2	p.Gly12Ser	c.34G>A	3997	1.822	46 %
	chr3:178936082	*PIK3CA*	NM_006218.2	Missense	9	p.Gly542Lys	c.1624 G>A	3934	334	8 %
	chr17:7577551	*TP53*	NM_000546.5	Missense	7	p.Gly244Ser	c.730G>A	4000	1.632	41 %
CRC28	chr17:7577559	*TP53*	NM_000546.5	Missense	7	p.Ser241Phe	c.722C>T	2123	587	28 %
CRC30	chr12:25398284	*KRAS*	NM_033360.3	Missense	2	p.Gly12Val	c.35G>T	3486	346	10 %
	chr17:7577094	*TP53*	NM_000546.5	Missense	8	p.Arg282Trp	c.844C>T	580	220	38 %
CRC31	chr12:25378562	*KRAS*	NM_033360.3	Missense	4	p.Ala146Thr	c.436G>A	4000	1.003	25 %
	chr12:25398262	*KRAS*	NM_033360.3	Missense	2	p.Leu19Phe	c.57G>T	3175	683	22 %
CRC32	chr12:25398284	*KRAS*	NM_033360.3	Missense	2	p.Gly12Asp	c.35G>A	2595	994	38 %
	chr3:178936091	*PIK3CA*	NM_006218.2	Missense	9	p.Glu545Lys	c.1633G>A	2186	514	24 %
	chr17:7578388	*TP53*	NM_000546.5	Missense	5	p.Arg181His	c.542G>A	1668	541	32 %
	chr17:7578461	*TP53*	NM_000546.5	Missense	5	p.Val157Leu	c.469G>C	1665	1.038	62 %
CRC33	chr12:25398282	*KRAS*	NM_033360.3	Missense	2	p.Gly13Cys	c.37G>T	3997	1.037	26 %
CRC34	chr17:7577022	*TP53*	NM_000546.5	Nonsense	8	p.Arg306Ter	c.916C>T	926	279	30 %
CRC35	chr17:7577120	*TP53*	NM_000546.5	Missense	8	p.Arg273His	c.818G>A	520	260	50 %
CRC36	chr17:7577538	*TP53*	NM_000546.5	Missense	7	p.Arg248Gln	c.743G>A	1240	567	45 %
CRC38	chr12:25378561	*KRAS*	NM_033360.3	Missense	4	p.Ala146Val	c.437C>T	3100	352	11 %
	chr17:7577538	*TP53*	NM_000546.5	Missense	7	p.Arg248Gln	c.743G>A	1387	201	14 %
CRC39	chr12:25398284	*KRAS*	NM_033360.3	Missense	2	p.Gly12Val	c.35G>T	3088	429	14 %
	chr3:178936082	*PIK3CA*	NM_006218.2	Missense	9	p.Gly542Lys	c.1624 G>A	3759	218	6 %
CRC40	chr7:140453148	*BRAF*	NM_004333.4	Missense	15	p.Gly596Val	c.1787G>T	3915	647	17 %
	chr12:25398284	*KRAS*	NM_033360.3	Missense	2	p.Gly12Asp	c.35G>A	3979	606	15 %
	chr17:7578406	*TP53*	NM_000546.5	Missense	5	p.Arg175His	c.524G>A	820	220	27 %
CRC41	chr12:25398284	*KRAS*	NM_033360.3	Missense	2	p.Gly12Asp	c.35G>A	3309	756	23 %
CRC42	chr4:153249348	*FBXW7*	NM_033632.3	Missense	8	p.Arg465His	c.1394G>A	2213	624	28 %
	chr12:25398285	*KRAS*	NM_033360.3	Missense	2	p.Gly12Ser	c.34G>A	2981	246	8 %
CRC43	chr12:25398284	*KRAS*	NM_033360.3	Missense	2	p.Gly12Val	c.35G>T	3972	1.210	30 %
	chr18:48591892	*SMAD4*	NM_005359.5	Missense	9	p.Gly352Val	c.1055G>T	3997	377	9 %
CRC44	chr12:25398285	*KRAS*	NM_033360.3	Missense	2	p.Gly12Ser	c.34G>A	5555	2.817	51 %
	chr3:178936082	*PIK3CA*	NM_006218.2	Missense	9	p.Gly542Lys	c.1624 G>A	3813	790	21 %
CRC45	chr17:7577081	*TP53*	NM_000546.5	Missense	8	p.Glu286Gly	c.857A>G	1722	843	49 %
CRC46	chr14:105246551	*AKT*	NM_0001014431.1	Missense	4	p.Glu17Lys	c.49G>A	700	21	3 %
	chr12:25399291	*KRAS*	NM_033360.3	Missense	2	p.Gly13Asp	c.38G>A	4000	243	6 %
CRC47	chr12:25398284	*KRAS*	NM_033360.3	Missense	2	p.Gly12Asp	c.35G>A	5059	476	9 %
	chr3:178936095	*PIK3CA*	NM_006218.2	Missense	9	p.Gln546Arg	c.1637A>G	4078	623	15 %
	chr18:48591918	*SMAD4*	NM_005359.5	Missense	9	p.Arg361Cys	c.1081C>T	3348	551	16 %
CRC49	chr12:25398284	*KRAS*	NM_033360.3	Missense	2	p.Gly12Asp	c.35G>A	2185	1.198	55 %
CRC51	chr12:25398284	*KRAS*	NM_033360.3	Missense	2	p.Gly12Phe	c.34_35delGGinsTT	3707	1.380	37 %
	chr7:116411923	*MET*	NM_001127500.1	Missense	14	p.Arg988Cys	c.2962C>T	2473	893	36 %
	chr17:7578406	*TP53*	NM_000546.5	Missense	5	p.Arg175His	c.524G>A	2473	893	36 %
CRC53	chr12:25398284	*KRAS*	NM_033360.3	Missense	2	p.Gly12Val	c.35G>T	3972	1.210	30 %
	chr3:178952085	*PIK3CA*	NM_006218.2	Missense	20	p.His1047Arg	c.3140A>G	3270	1.921	59 %
CRC54	chr12:25398284	*KRAS*	NM_033360.3	Missense	2	p.Gly12Asp	c.35G>A	4000	1.985	50 %
	chr17:7577539	*TP53*	NM_000546.5	Missense	7	p.Arg248Trp	c.742C>T	1219	352	29 %
CRC55	chr12:25398284	*KRAS*	NM_033360.3	Missense	2	p.Gly12Asp	c.35G>A	2713	447	16 %

	chr3:178936091	*PIK3CA*	NM_006218.2	Missense	9	p.Glu545Lys	c.1633G>A	3519	557	16 %
CRC57	chr17:7577094	*TP53*	NM_000546.5	Missense	8	p.Arg282Trp	c.844C>T	2827	2.227	79 %
	chr4:153249446	*FBXW7*	NM_033632.3	Missense	11	p.Ser582Leu	c.1745C>T	6903	1.631	24 %
CRC58	chr12:25378562	*KRAS*	NM_033360.3	Missense	4	p.Ala146Val	c.437C>T	3503	1.575	45 %
	chr17:7578263	*TP53*	NM_000546.5	Nonsense	6	p.Arg196Ter	c.586C>T	2290	771	34 %
CRC59	chr17:7577538	*TP53*	NM_000546.5	Missense	7	p.Arg248Gln	c.743G>A	2905	652	22 %
	chr4:153249348	*FBXW7*	NM_033632.3	Missense	8	p.Arg465His	c.1394G>A	4444	1.148	26 %
CRC60	chr12:25398284	*KRAS*	NM_033360.3	Missense	2	p.Gly12Val	c.35G>T	5064	1.715	34 %
	chr18:48591918	*SMAD4*	NM_005359.5	Missense	9	p.Arg361Cys	c.1081C>T	5582	313	6 %
	chr17:7578406	*TP53*	NM_000546.5	Missense	5	p.Arg175His	c.524G>A	2198	1.071	49 %
	chr3:41266136	*CTNNB1*	NM_001904.3	Missense	3	p.Ser45Pro	c.133T>C	1961	812	41 %
CRC61	chr12:25378562	*KRAS*	NM_033360.3	Missense	4	p.Ala146Thr	c.436G>A	3232	653	20 %
	chr10:89717769	*PTEN*	NM_000314.4	Deletion	7	p.Lys267fs	c.795del	3202	917	29 %
	chr10:89720852	*PTEN*	NM_000314.4	Missense	8	p.Arg335Ter	c.1003C>T	1932	505	26 %
CRC62	chr12:25398284	*KRAS*	NM_033360.3	Missense	2	p.Gly12Asp	c.35G>A	6260	908	15 %
CRC63	chr17:7578406	*TP53*	NM_000546.5	Missense	5	p.Arg175His	c.524G>A	2317	710	31 %
CRC64	chr12:25398284	*KRAS*	NM_033360.3	Missense	2	p.Gly12Val	c.35G>T	7456	2.076	28 %
CRC65	chr12:25398284	*KRAS*	NM_033360.3	Missense	2	p.Gly12Asp	c.35G>A	8743	3.635	42 %
	chr17:7577509	*TP53*	NM_000546.5	Missense	7	p.Glu258Lys	c.772G>A	2227	1.475	66 %
CRC67	chr12:25399291	*KRAS*	NM_033360.3	Missense	2	p.Gly13Asp	c.38G>A	1043	120	12 %
CRC68	chr17:7578550	*TP53*	NM_000546.5	Missense	5	p.Ser127Phe	c.380C>T	893	444	50 %
CRC69	chr17:7577120	*TP53*	NM_000546.5	Missense	8	p.Arg273His	c.818G>A	512	236	46 %
CRC70	chr12:25398284	*KRAS*	NM_033360.3	Missense	2	p.Gly12Asp	c.35G>A	4342	1.861	43 %
	chr18:48591918	*SMAD4*	NM_005359.5	Missense	9	p.Arg361Cys	c.1081C>T	2151	455	21 %
CRC71	chr17:7574018	*TP53*	NM_000546.5	Missense	10	p.Arg337Cys	c.1009C>T	503	65	13 %
	chr17:7578437	*TP53*	NM_000546.5	Nonsense	5	p.Gln165Ter	c.493C>T	503	65	13 %
CRC72	chr12:25378561	*KRAS*	NM_033360.3	Missense	4	p.Ala146Val	c.437C>T	3852	1.551	40 %
	chr17:7578406	*TP53*	NM_000546.5	Missense	5	p.Arg175His	c.524G>A	900	219	24 %
CRC73	chr7:140453136	*BRAF*	NM_004333.4	Missense	15	p.Val600Glu	c.1799T>A	1926	572	30 %
	chr10:89711907	*PTEN*	NM_000314.4	Missense	6	p.Tyr177His	c.529T>C	1756	556	32 %
CRC75	chr12:25398284	*KRAS*	NM_033360.3	Missense	2	p.Gly12Asp	c.35G>A	3670	1.084	30 %
	chr17:577548	*TP53*	NM_000546.5	Missense	7	p.Gly245Ser	c.733G>A	3999	523	13 %
	chr7:140453136	*BRAF*	NM_004333.4	Missense	15	p.Val600Glu	c.1799T>A	2207	619	28 %
CRC76	chr17:7577580	*TP53*	NM_000546.5	Missense	7	p.Tyr234Cys	c.701A>G	3916	1.013	26 %
	chr17:7578388	*TP53*	NM_000546.5	Missense	5	p.Arg181Pro	c.542G>C	2798	493	18 %
CRC79	chr3:41266137	*CTNNB1*	NM_001904.3	Missense	3	p.Ser45Phe	c.134C>T	2738	1.684	62 %
	chr12:25399291	*KRAS*	NM_033360.3	Missense	2	p.Gly13Asp	c.38G>A	5040	1.716	34 %
CRC80	chr18:48591918	*SMAD4*	NM_005359.5	Missense	9	p.Arg361His	c.1082G>A	5362	1.763	33 %
	chr17:7577538	*TP53*	NM_000546.5	Missense	7	p.Arg248Gln	c.743G>A	10196	3.395	33 %
	chr12:25398284	*KRAS*	NM_033360.3	Missense	2	p.Gly12Asp	c.35G>A	3843	656	17 %
CRC81	chr18:48591901	*SMAD4*	NM_005359.5	Missense	9	p.Asp355Val	c.1064A>T	2709	266	10 %
	chr17:7578212	*TP53*	NM_000546.5	Nonsense	6	p.Arg213Ter	c.637C>T	2974	272	9 %
	chr7:140453132	*BRAF*	NM_004333.4	Missense	15	p.Lys601Asn	c.1803A>C	1289	161	12 %
CRC84	chr1:115256529	*NRAS*	NM_002524.4	Missense	3	p.Gln61Arg	c.182A>G	2955	327	11 %
	chr17:7577124	*TP53*	NM_000546.5	Missense	8	p.Val272Leu	c.814G>T	1156	147	13 %
CRC82	chr12:25398284	*KRAS*	NM_033360.3	Missense	2	p.Gly12Asp	c.35G>A	2514	1.051	42 %

CRC85	chr7:140453136	*BRAF*	NM_004333.4	Missense	15	p.Val600Glu	c.1799T>A	2078	291	14 %
	chr3:178936082	*PIK3CA*	NM_006218.2	Missense	9	p.Gly542Lys	c.1624 G>A	3964	849	21 %
CRC86	chr15:66727455	*MAP2K1*	NM_002755.3	Missense	2	p.Lys57Asn	c.171G>C	1408	169	12 %
	chr18:48591918	*SMAD4*	NM_005359.5	Missense	9	p.Arg361His	c.1082G>A	3722	599	16 %
CRC87	chr12:25399291	*KRAS*	NM_033360.3	Missense	2	p.Gly13Asp	c.38G>A	4564	500	11 %
	chr17:7577094	*TP53*	NM_000546.5	Missense	8	p.Arg282Trp	c.844C>T	876	134	15 %
CRC88	chr4:153258983	*FBXW7*	NM_033632.3	Nonsense	4	p.Arg278Ter	c.832C>T	5498	547	10 %
	chr12:25398285	*KRAS*	NM_033360.3	Missense	2	p.Gly12Ser	c.34G>A	6281	1.442	23 %
	chr17:7577563	*TP53*	NM_000546.5	Missense	7	p.Asn239Ile	c.716A>T	3477	557	16 %
	chr17:7577563	*TP53*	NM_000546.5	Missense	7	p.Ser240Gly	c.718A>G	3477	557	16 %
CRC89	chr12:25398284	*KRAS*	NM_033360.3	Missense	2	p.Gly12Ser	c.34G>A	8221	1.366	17 %
CRC90	chr17:7578272	*TP53*	NM_000546.5	Missense	6	p.His193Tyr	c.577C>T	1911	233	12 %
CRC91	chr7:140453136	*BRAF*	NM_004333.4	Missense	15	p.Val600Glu	c.1799T>A	3104	686	22 %
	chr18:48591918	*SMAD4*	NM_005359.5	Missense	9	p.Arg361Cys	c.1081C>T	2253	460	20 %
CRC92	chr7:140453136	*BRAF*	NM_004333.4	Missense	15	p.Val600Glu	c.1799T>A	2077	142	7 %
	chr17:7577120	*TP53*	NM_000546.5	Missense	8	p.Arg273His	c.818G>A	2077	142	7 %
CRC94	chr17:7578529	*TP53*	NM_000546.5	Missense	5	p.Phe134Cys	c.401T>G	3395	1.329	39 %
CRC95	chr12:25398284	*KRAS*	NM_033360.3	Missense	2	p.Gly12Asp	c.35G>A	1386	495	36 %
	chr17:7577094	*TP53*	NM_000546.5	Missense	8	p.Arg282Trp	c.844C>T	821	226	28 %
CRC97	chr9:139399362	*NOTCH*	NM_017617.3	Missense	26	p.Arg1594Gln	c.4781G>A	560	252	45 %
CRC98	chr17:7577140	*TP53*	NM_000546.5	Frameshift	8	p.Gly266fs	c.797delG	905	445	49 %
CRC99	chr12:25398284	*KRAS*	NM_033360.3	Missense	2	p.Gly12Val	c.35G>T	3984	1.423	36 %
	chr17:7577094	*TP53*	NM_000546.5	Missense	8	p.Arg282Trp	c.844C>T	1758	875	50 %
CRC100	chr18:48591918	*SMAD4*	NM_005359.5	Missense	9	p.Arg361His	c.1082G>A	2325	745	32 %
	chr17:7578266	*TP53*	NM_000546.5	Missense	6	p.Ile195Phe	c.583A>T	689	219	32 %

CDS- Coding sequence nomenclature; Total Coverage- Total reads in the genomic region; Variant Coverage- Total variant reads; VAF- Variant allele frequency (%).

© 2019 by the authors. Licensee MDPI Basel Switzerland. This article is an open access article distributed under the terms and conditions of the Creative Commons Attribution (CC BY) license (http://creativecommons.org/licenses/by/4.0/).
